# Can motorcycle riding in Australia really be that thermally stressful?

**DOI:** 10.1186/2046-7648-4-S1-A20

**Published:** 2015-09-14

**Authors:** Liz de Rome, Elizabeth A Taylor, Rodney J Croft, Julie Brown, Nigel AS Taylor

**Affiliations:** 1 Neuroscience Research Australia, Sydney and the Centre for Human and Applied Physiology, School of Medicine, University of Wollongong, Wollongong, Australia

## Introduction

Personal protective clothing and equipment, when used during moderate to heavy workloads, can push individuals to the limits of physiological regulation. What is less certain is the physiological strain imposed by appropriately protective ensembles when riding a motorcycle, and whether or not one need even consider the impact of that strain. Nevertheless, evidence indicates that thermal discomfort is a key disincentive to the wearing of motorcycle protective clothing in hot weather. Therefore, the purpose of this investigation was to quantify thermal and cardiovascular strain during a simulated urban, motorcycle ride, conducted under laboratory conditions.

## Methods

Twelve males were investigated on four occasions across three thermal environments: 25 °C (water vapour pressure 1.3 kPa), 30 °C (1.7 kPa) and 35 °C (2.25 kPa). Each trial was comprised of three, 30-min stages: 25 min cycling (30 W) plus 5 min rest. Heart rates, auditory-canal and mean skin temperatures were measured. Subjects were also exposed to an overhead radiant heat load and a 30 km.h^-1 ^headwind. Clothing was modified between full accident protection (helmet, jacket, trousers, gloves, boots; ventilation ports closed) and an unprotected state (helmet, gloves, boots, jeans, long-sleeve t-shirt), with both conditions tested at 25 °C, and the full protective ensemble also investigated at 30 °C and 35 °C.

## Results

Increasing rider protection at 25 °C did not modify auditory-canal temperature (*P *> 0.05), but mean skin temperatures (2°C) and steady-state heart rates were increased (12 beats.min^-1^; both *P*<0.05) with increased protection. Increments in air temperature reduced the skin-air temperature gradient (*P*<0.05) and dry heat loss. Consequently, at 35 °C, auditory-canal temperature rose at 0.02°C.min^-1 ^(SD 0.005), deviating from all trials (*P*<0.05), and the thresholds for moderate (>38.5 °C) and profound hyperthermia (>40.0 °C) were predicted to occur within 105 min (SD 20.6) and 180 min (SD 33.0). The latter might eventuate in ~10 h at 30 °C, but would not occur at 25 °C. Thermal discomfort increased sequentially in the 35°C trial, averaging between "uncomfortable" and "very uncomfortable".

## Discussion

The principal outcomes from this experiment were that, within air temperatures that approximated deep-body temperature, as may be encountered during an Australian summer, urban motorcyclists would be likely to approach profound hyperthermia and potentially debilitating central cardiovascular strain within 3 h, and earlier in sedentary riders. However, at air temperatures of 25^o^and 30 °C, neither of those outcomes would be likely. What remains uncertain is whether or not the level of physiological strain encountered at 35 °C would have an adverse impact upon cognitive function, perhaps through reducing cerebral blood flow, which may, in turn, elevate the risk of motorcycle accidents. This possibility forms the basis of ongoing research.

## Conclusion

This experiment was designed to evaluate the physiological significance of motorcycle impact-protective ensembles under close to worst-case conditions. The outcomes indicate that greater design attention is required to enhance dry and evaporative heat dissipation from clothing intended for summer use in hotter climates, but without compromising injury protection.

